# Identification of MAP3K4 as a novel regulation factor of hepatic lipid metabolism in non-alcoholic fatty liver disease

**DOI:** 10.1186/s12967-022-03734-8

**Published:** 2022-11-14

**Authors:** Zhiyong He, Yangyang Bin, Guangshun Chen, Qiang Li, Wenling Fan, Yongqiang Ma, Junfang Yi, Xiaohua Luo, Zhi Tan, Jiequn Li

**Affiliations:** 1 Department of Liver Transplant, The Second Xiangya Hospital of Central South University, 139 Renmin Road, Changsha, Hunan 410011 People’s Republic of China; 2 National Clinical Research Center for Metabolic Diseases, The Second Xiangya Hospital of Central South University, Changsha, 410011 China; 3 Department of General Surgery, Xiangtan Central Hospital, Xiangtan, 411100 China; 4 Department of Gastroenterology, The First Hospital of Changsha, Changsha, 410005 China

**Keywords:** Non-alcoholic fatty liver disease, Lipid droplets, Transcriptomics analysis, *MAP3K4*

## Abstract

**Background:**

Non-alcoholic fatty liver disease (NAFLD) is a metabolic disorder with abnormal lipid metabolism. The present study was to identify regulatory genes related to lipid droplets (LDs) abnormal accumulation in NAFLD.

**Methods:**

transcriptomic analysis and bioinformatics analysis (GEO database) were used to identify potential genes in abnormal lipid metabolism of NAFLD. A candidate gene *MAP3K4* expression were detected by immunohistochemistry staining in NAFLD and controls. RNA interference and immunoblotting were used to verify the roles of *MAP3K4* in the formation of hepatic LDs.

**Results:**

A total of 134 candidate genes were screened, including 44 up-regulated genes and 90 down-regulated genes. 29 genes in the protein–protein interaction (PPI) were selected as hub genes, including *MAP3K4*. The expression levels of *MAP3K4* were positively correlated with NAFLD activity score (r = 0.702, p = 0.002). Furthermore, we found a positive correlation of MAP3K4 expression with serum total cholesterol (r = 0.564, p = 0.023), uric acid levels (r = 0.520, p = 0.039), and body mass index (r = 0.574, p = 0.020). Downregulation of *MAP3K4* decreased LDs accumulation in HepG2 cells and reduced the expression of CGI-58 and Plin-2 by imbibition of JNK and group IVA cytosolic phospholipase A2 (cPLA2) activation.

**Conclusion:**

The study revealed a number of regulatory genes related to hepatic lipid metabolism of NAFLD, and demonstrated that *MAP3K4* played a pivotal role in the hepatic lipogenesis of NAFLD.

**Supplementary Information:**

The online version contains supplementary material available at 10.1186/s12967-022-03734-8.

## Introduction

Nonalcoholic fatty liver disease (NAFLD) is a broad-spectrum term used to generalize non-alcoholic fatty liver simple steatosis and non-alcoholic steatohepatitis (NASH). Simple steatosis is the initial stage within the spectrum of NAFLD, which progresses to NASH and increases the risk of developing fibrosis, cirrhosis, and hepatocellular carcinoma [1, 2]. NAFLD has affected 10 to 48 percent of the general population in different countries of the world [3–5]. As the epidemics of obesity and type 2 diabetes mellitus (T2DM), the incidence and prevalence of NAFLD which is the most common cause of adult liver disease across various countries are growing over time [3].

It is well-accepted that non-alcoholic hepatic steatosis is primarily caused by insulin resistance (IR) [1, 6–8]. Mitochondrial dysfunction has been shown to be related with the occurrence of hepatic IR of NAFLD patients [9]. In addition, free fatty acid (FFA)-induced lipid toxicity and the inflammatory response are two main mechanisms that cause hepatic IR in NAFLD [7]. The mitochondrial dysfunction also contributes to fat accumulation and liver damage by oxidative stress, increases reactive oxygen species generation. NAFLD is also closely related to inflammation and oxidative stress [10, 11]. It is well recognized that oxidative stress causes cellular dysfunction and is a pathophysiologic cause of NAFLD. Oxidative stress develops when the production of reactive oxygen species surpasses the ability of antioxidants to detoxify them, which causes harm to normal lipid metabolism [12]. However, there is currently no established single-drug or combined therapy. There are numerous studies on the importance of nano-antioxidants in the prevention and treatment of liver disorders, including hepatic ischemia–reperfusion injury, viral hepatitis, hepatocellular cancer and viral fibrosis [13–16]. Other treatments of targeting reactive oxygen species have been shown to play an protective to injury of rat hepatocytes [17, 18]. Furthermore, an appealing therapeutic approach for the management of NAFLD involves targeting mitochondria. Antioxidants targeting mitochondrial ^•^O_2_^−^/H_2_O_2_, for instance, have the potential to treat NAFLD by counteracting liver inflammation [19].

The increased fatty acids absorption, increased de novo lipogenesis, and impairment in export and oxidation of fatty acids induced intracellular lipid accumulation and lipid droplets (LDs) formation, which is the histological feature of NAFLD in the liver [20]. Genetic studies also provided clues to the study of hepatic lipogenesis of NAFLD. Genome-wide association studies (GWAS) have shown that genetic and epigenetic factors acted as regulators in NAFLD. A major genetic factor I148M PNPLA3 variation, a triglyceride hydrolase, increased susceptibility to NAFLD [21]. Transcriptome and single-cell sequencing studies have also been applied to study the pathogenesis of NAFLD. Numerous signaling pathways and genes have been shown to be involved in the pathogenesis of NAFLD. TLR4-dependent inflammatory factor release pathway, PI3K/Akt signaling pathway and TGF-β/SMAD3-signaling pathway play an important roles in the occurrence of NAFLD [22–24]. Besides, *CYP2E1* has been shown to be involved in LDs formation in NAFLD. Overexpression of *CYP2E1 *in vivo or in vitro, the development of hepatic IR was promoted by JNK activation [25, 26]. *CYP3A* activity was decreased in hepatoma cell models, high fat diet-induced mice and NAFLD patients [27]. Meanwhile, variants of *GCKR, TM6SF2* and *MBOAT7* genes have been shown significant contributions to the occurrence of NAFLD [28]. However, the pathogenesis of NAFLD is not yet completely understood.

In the current study, we performed bioinformatics analysis to identify 134 overlapping genes by analyzing the differential expression gene from the GSE159676 datasets and our RNAseq data and then identified five MCODE (Molecular Complex Detection) modules and 29 hub genes. In one of these five modules, four of six hub genes has been reported to be associated with lipid metabolism [29–32]*.* These four hub genes were densely connected with Mitogen-activated protein kinase kinase kinase 4 (*MAP3K4*). However, the role of *MAP3K4* in lipid metabolism of NAFLD has not been reported. The present study focused on *MAP3K4* and explored its role and potential mechanism in NAFLD*.*

## Materials and methods

### Clinical data and samples

Nine control samples and seven patients with histologically confirmed NAFLD were included. Control liver samples were collected from normal liver tissues of hepatic hemangioma resection. Histological features were assessed using NAFLD activity score (NAS) [33]. Two professional pathologists who were blinded to the clinical data performed a histological assessment of liver specimens. The ethics committee of the Second Xiangya Hospital of Central South University granted ethical permission for this experiment. Written informed consent was obtained from all participants. The clinical characteristics between NAFLD patients and controls were displayed in Additional file 1.

### Hepatic transcriptome

Hepatic transcriptome analysis was performed by the Next Generation Sequence of RNA extracts from liver specimens as previously described [34–36]. Briefly, total RNA was isolated using the Illustra RNAspin Mini Kit (GE Healthcare, United States). cDNA libraries and sequencing library were generated by TruSeq Stranded Total RNA kit (Illumina Inc.) according to the manufacturer’s instructions. RNA sequencing was performed via Illumina HiSeq2000 high-throughput sequencing system (Illumina Inc.). Adaptor sequences and low-quality reads were removed. Quality control of the raw fastq files was performed using the software Fastq-mcf v1.0.3-r152. Reads were mapped to the annotated Human genome (Human GRCh38/hg38) in Ensembl database. Subsequently, the gene expression profiling, differentially expressed genes, and differentially expressed transcripts were calculated.

### Immunohistochemistry

Liver tissues were fixed with paraformaldehyde and embedded in paraffin. The paraffin-embedded tissues were cut into 5 µm slices. After deparaffinization, the slices were incubated 30 min with 10% of goat serum in TBS (50 mM Tris–HCl, pH 7.4, 150 mM NaCl) at room temperature. Subsequently, the tissues were incubated with anti-*MAP3K4* antibody (Sigma, United States). After washed with TBS, incubated with the HRP-conjugated secondary antibody (Jackson Immunoresearch, United States), Then, sections were treated with DAB. All stained sections were observed and imaged on Olympus BX41microscope (Olympus, Tokyo, Japan).

### RNA interference

The oligos complementary for *MAP3K4* RNA and nontarget oligos were synthesized by Gene Pharma Co. Ltd. (Sangon Biotech, China). The target sequence siRNA #1 for human *MAP3K4* target sequence is 5'-GAGTCCTGAATCTGATCTAGA-3', and siRNA #2 target sequence is 5'-GTCCAGCAGATCGTTTAAAGT-3'. According to the instructions provided by the manufacturer, HepG2 cells were transfected by the oligos for *MAP3K4* interference and control oligo via Lipofectamine 2000 Transfection Reagent (Invitrogen, USA).

### BODIPY and immunofluorescence staining

The BODIPY staining was performed as previously described [35]. After 36 h transfection with siRNA, the cells were treated with oleic acid (OA) + palmitic acid (PA) for 12 h [34]. The cells were washed with PBS twice and fixed in 4% of paraformaldehyde for 15 min. Then, the cells were permeabilized with PBST (PBS + 0.1% Triton X-100). Subsequently, the cells were blocked with 5% BSA/PBS for 30 min and washed with PBS and incubated in BODIPY 493/503 staining solution (Sigma, United States) for 15 min at 37 °C. Finally, the nuclei were counterstained with DAPI (Invitrogen, United States) for 2 min. All stained sections were observed and imaged on a Zeiss 880 confocal microscope.

### Immunoblotting

CGI-58 and Plin-2 antibodies were obtained from Abcam. β-actin, Phospho-JNK (Thr183, Tyr185), JNK, Phospho-S505-cytosolic phospholipase A2 (cPLA2), and cPLA2 antibodies were purchased from Cell Signaling Technology Co. Lysis buffer (2% SDS, 62.5 mM Tris–HCl pH 6.8, and 10% glycerol) was used to lyse the cells. Subsequently, 20 μg protein of each sample was electrophoresed and separated by SDS-PAGE. Then, the proteins were transferred to PVDF membranes (Millipore Corporation, United States). The membrane was incubated in 5% of defatted milk for 1 h at room temperature. The membrane was incubated with the primary antibodies overnight at 4 °C. After incubation, the membrane was washed in PBST three times and subsequently incubated with HRP-conjugated secondary antibodies. Immobilon Western Chemiluminescent HRP substrate (Millipore Corporation, United States) was used to obtain visualization of target protein bands.

### Biochemical analysis

According to the manufacturer’s instructions, we detect aspartate aminotransferase (AST) enzyme activity using aspartate aminotransferase activity assay kit (ab105135) and alanine aminotransferase (ALT) enzyme activity using an alanine transaminase activity assay kit (Colorimetric/Fluorometric ab105134).

### Data handling and statistical analysis

KEGG pathway enrichment analysis and GO enrichment analysis were performed using Metascape. The database (GSE159676) was obtained from National Center for Biotechnology Information (NCBI). Protein–protein interaction (PPI) enrichment analysis was carried out by the Cytoscape software. When the network contained between 3 and 500 proteins, the MCODE algorithm was further used to identify the densely connected components of the network. Then, MCODE modules were detected from the PPI network using MCODE algorithm [37]. Hub genes were identified using MCODE algorithm and Betweenness algorithm of plug-in CytoHubba in Cytoscape, version 3.9.1. All data were represented as means ± standard error of the mean (SEM) using Prism 8.3.0 software. The statistical significance of the difference between groups was determined using Student’s t-test in Prism 8.3.0 software, with 0.05 as the cutoff for statistical significance.

## Results

### Transcriptome analysis and bioinformatics analysis identified a series of metabolism-related pathways and genes involved in NAFLD

**Fig. 1:**
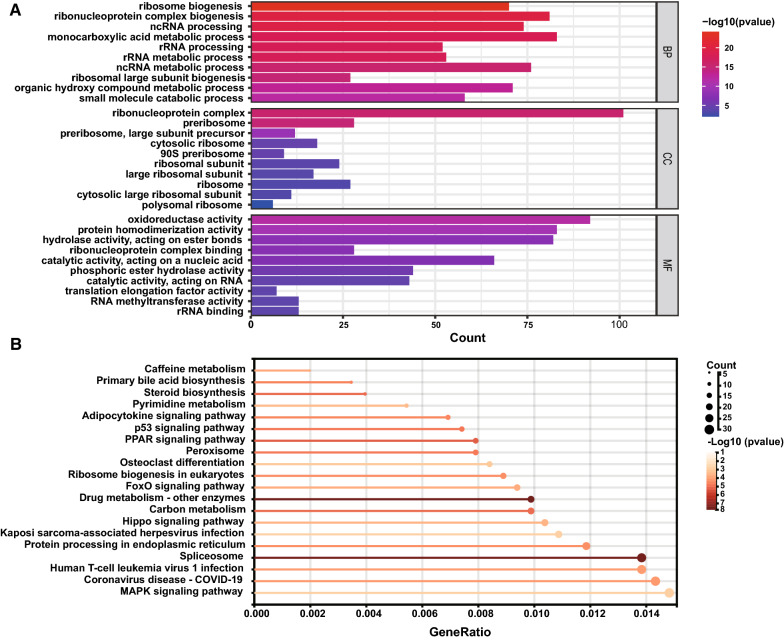
Results of GO and KEGG enrichment on differentially expressed genes from the RNA-seq data. (**A**) The top 30 enriched GO terms of RNA-seq related to NAFLD. (**B**) The top 20 significantly KEGG pathways of RNA-seq related to NAFLD with horizontal lollipops

To further explore the underlying regulations of lipid metabolism, transcriptomic analysis was carried out on liver specimens from seven patients with NAFLD and nine controls (Additional file 2). We found that the 1014 increased expression genes and 1010 decreased expression genes. Next, the KEGG and GO enrichment analyses were performed in Metascape (Additional file 3; Additional file 4). The top-ranked KEGG pathways and GO enrichment terms for differentially expressed genes were presented (Fig. 1A, )B). Together, these results demonstrated that differentially expressed genes enriched in Drug metabolism-other enzymes, MAPK signaling pathway, PPAR signaling pathway, Carbon metabolism, Steroid biosynthesis, and Spliceosome, among others accordance with their *p*-values. It has been found that many of these pathways are crucial for lipid metabolism [38–40].

**Fig. 2:**
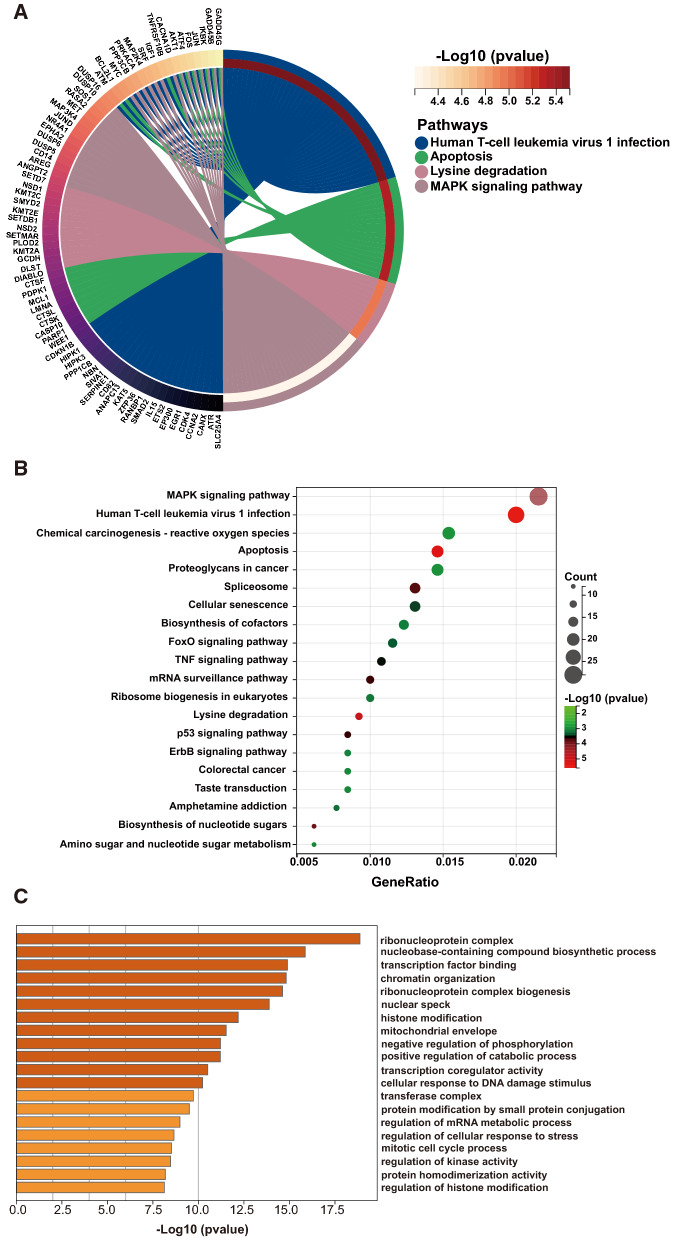
Results of GO and KEGG enrichment on differentially expressed genes from the RNA-seq data of GSE159676 datasets. **A** The top 4 KEGG pathways and genes in KEGG pathways with enrichment circle of GSE159676 datasets. **B** The top 20 significantly KEGG pathways related to NAFLD with bubble chart in GSE159676 datasets. **C** Bar graph with enriched biological process and pathways in GSE159676 datasets

To further screen out the candidate genes involved in lipid metabolism, we choose the GSE159676 datasets [41]. This database contained 7 representative NAFLD samples and 6 healthy controls. In the study, 496 up-regulated genes and 804 down-regulated genes were found. Differentially expressed genes were significantly enriched in Human T-cell leukemia virus infection, Apoptosis, Lysine degradation, and MAPK signaling pathway, among others accordance with their *p*-values (Fig. 2A). The top 20 KEGG pathways were identified, and many of these pathways have a connection to lipid metabolism, such as MAPK signaling pathway, apoptosis, and P53 signaling pathway [42–45] (Fig. 2B; Additional file 5). The GO enrichment analysis was also implemented to identify candidate genes (Fig. 2C; Additional file 6). The results of the GO analysis indicated that ribonucleoprotein complex was the most significant term related to NAFLD. It has been demonstrated that the ribonucleoprotein complex was crucial for lipid metabolism [46].

### The selection of candidate genes

**Fig. 3:**
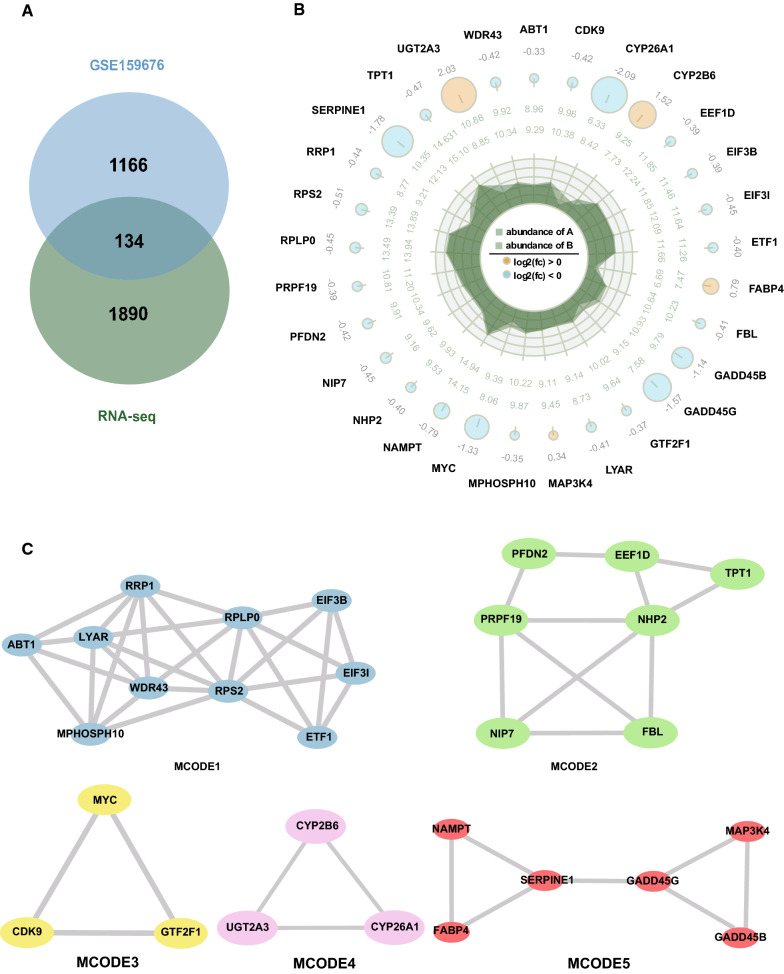
The comparative analysis of overlapping genes of RNA-seq data and GSE159676 datasets. **A** Venn diagram of the differentially expressed genes that appeared in both RNA-seq data and the GSE159676 datasets. **B** The expression of 29 hub genes on transcription of NAFLD patients and controls. The bigger circle, the greater the differential expression level. Out to inner numbers in circles represented log_2_FC of the expression level of group A and B. A represented NAFLD group and B represented control group. **C** The five most significant MCODE components from the PPI network of overlapping genes

To further identify candidate genes involved in lipid metabolism, we combined RNA-seq data and the GSE159676 datasets to analyze. 134 candidate genes were presented in the common lists. (Fig. 3A, Additional file 7). The PPI analysis of 134 genes were conducted by the STRING database and Cytoscape software to further understand protein interaction (Additional file 8). To determine which network nodes were closely connected, the MCODE algorithm was used. The five significant MCODE components were identified from the PPI network of overlapping genes using Metascape. We obtained 29 hub genes, including *MAP3K4*. The expression of these genes on transcription were shown in Fig. 3B. The five significant MCODE components were shown in Fig. 3C. In the MCODE 5 (Fig. 3C), four of six hub genes (*FABP4*, *SERPINE1*, *GADD45B* and *NAMPT*) has been reported to be associated with lipid metabolism [29–32]. Furthermore, all of these hub genes were densely connected with *MAP3K4* (Fig. 3C). Additionally, we obtained the top 10 hub genes using a common algorithm of plug-in CytoHubba, including *MAP3K4, MYC, FBL, NHP2, CCNA2, GADD45B, SERPINE1, EIF3B, ARF1, BCL2L1* (Additional file 9). Overlap analysis the above two list, *MAP3K4* was selected as a candidate gene. These findings suggested that the lipid metabolism of NAFLD might be regulated by *MAP3K4*. Therefore, we selected *MAP3K4* as the candidate gene for further verification.

### An increased expression of *MAP3K4* in liver tissues from NAFLD patients

**Fig. 4:**
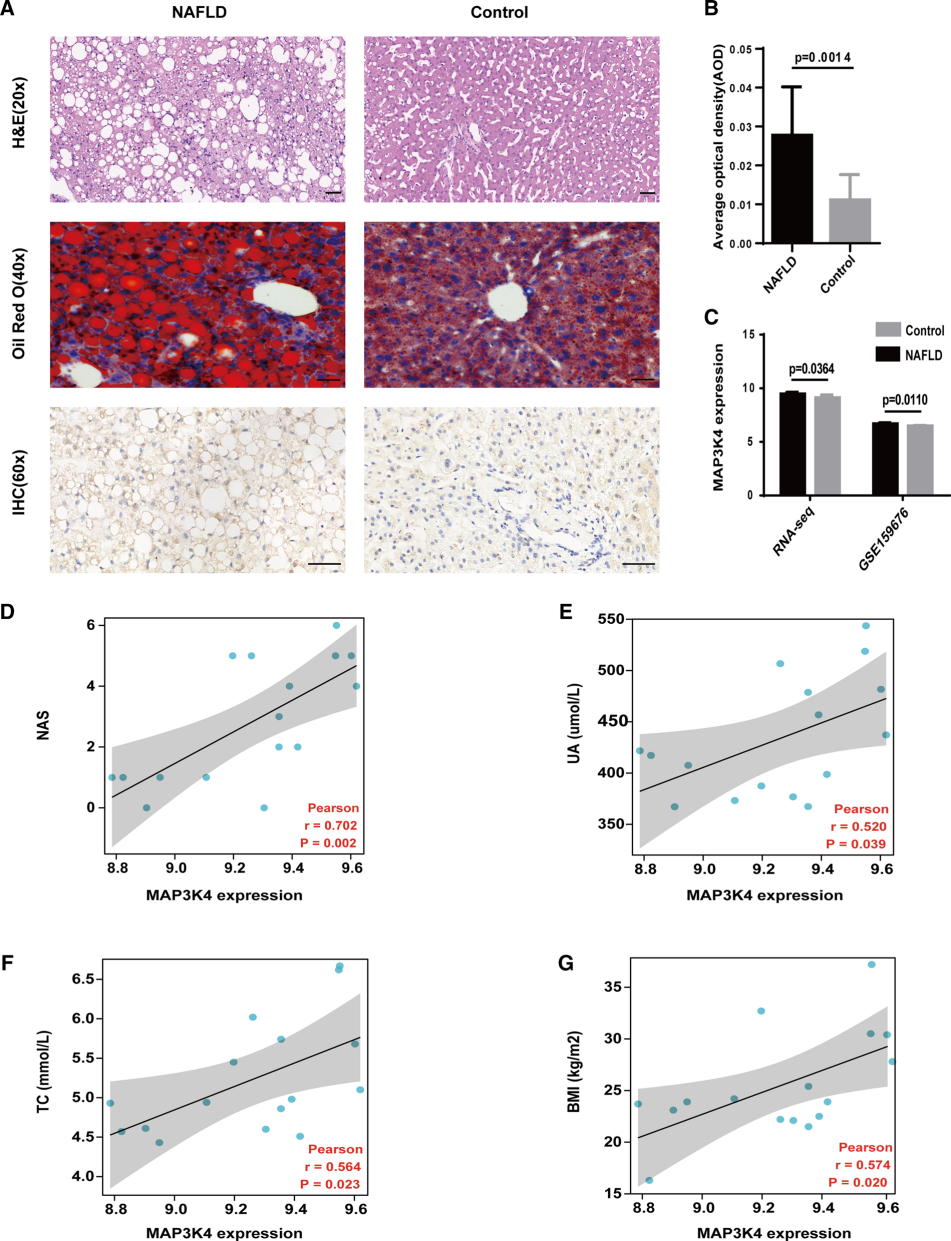
The expression of *MAP3K4* in liver tissue from NAFLD patients. **A** Representative images of H&E staining, Oil red O staining, and immunohistochemistry assay for MAP3K4. Scale bars = 50 μm. **B** Quantitative results of MAP3K4 expression (n = 7, 2–3 slides/patient). **C** mRNA relative levels of *MAP3K4* from RNA-seq data and the GSE159676 datasets. **D**–**G** Correlation analysis between the expression levels of MAP3K4 and NAS score, serum UA, TC levels and BMI. NAS, NAFLD activity score; UA, uric acid; TC, total cholesterol; BMI, body mass index

As shown in Fig. 4A, Oil Red O staining and H&E revealed that the NAFLD patients had an obvious cytoplasmic LDs accumulation and hepatocyte ballooning degeneration. Compared with the controls, the levels of MAP3K4 expression by immunohistochemical staining were significantly elevated in the liver tissues of NAFLD patients (Fig. 4B). Additionally, analysis of our RNA-seq data and GSE159676 datasets also indicated that patients with NAFLD had significantly higher levels of *MAP3K4* expression compared to controls (9.45 ± 0.17 *vs.* 9.11 ± 0.25 *p* = 0.0364) (Fig. 4C, Additional file 2). Furthermore, the expression levels MAP3K4 were positive correlation with NAS score (*r* = 0.702, *p* = 0.002) (Fig. 4D).

Pearson’s correlation analysis was used to investigate whether the alterations of clinical indicators of lipid metabolism correlated with the expression levels of MAP3K4. Our results showed *MAP3K4* expression levels were significantly correlated the serum uric acid (UA, *r* = 0.520, *p* = 0.039), total cholesterol (TC, *r* = 0.564, *p* = 0.023) levels and body mass index (BMI, r = 0.574, *p* = 0.020) (Fig. 4E–G). According to clinical practice guidelines by the Asian Pacific Association, TC and BMI were crucial in the diagnosis of NAFLD [47]. These findings suggested that *MAP3K4* might be play a crucial role in lipid metabolism in NAFLD patients.

### *MAP3K4 r*egulated the activation of JNK and cPLA2 in the biogenesis of LDs

**Fig. 5:**
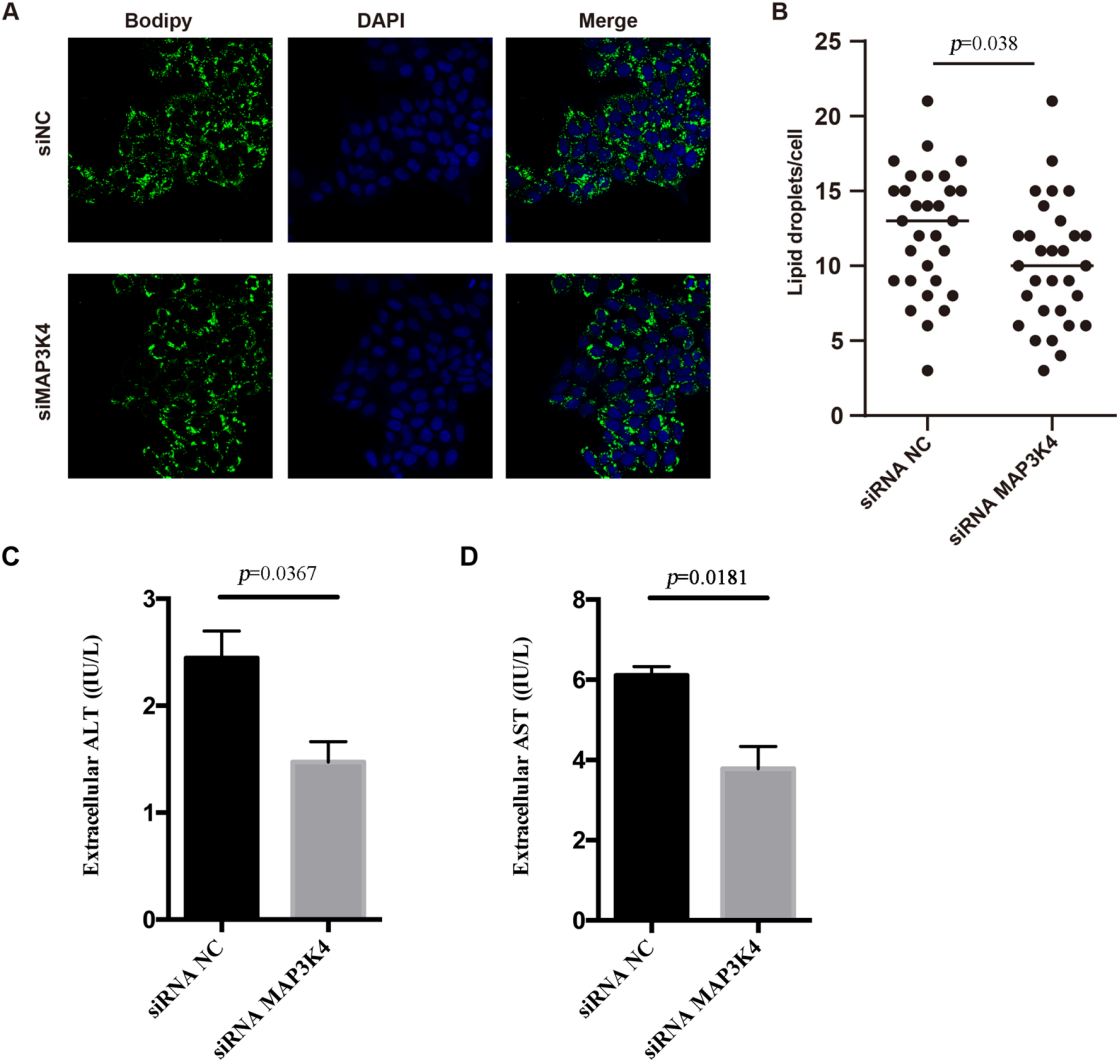
*MAP3K4* regulates LDs formation. **A** Representative images of HepG2 cells transfected with *MAP3K4* siRNA and control siRNA. BODIPY staining for LDs. DAPI staining for cell nuclear. **B** Quantification of lipid droplets per cell. **C** MAP3K4 regulates extracellular ALT in HepG2 cells **D** MAP3K4 regulates extracellular AST in HepG2 cells

**Fig. 6:**
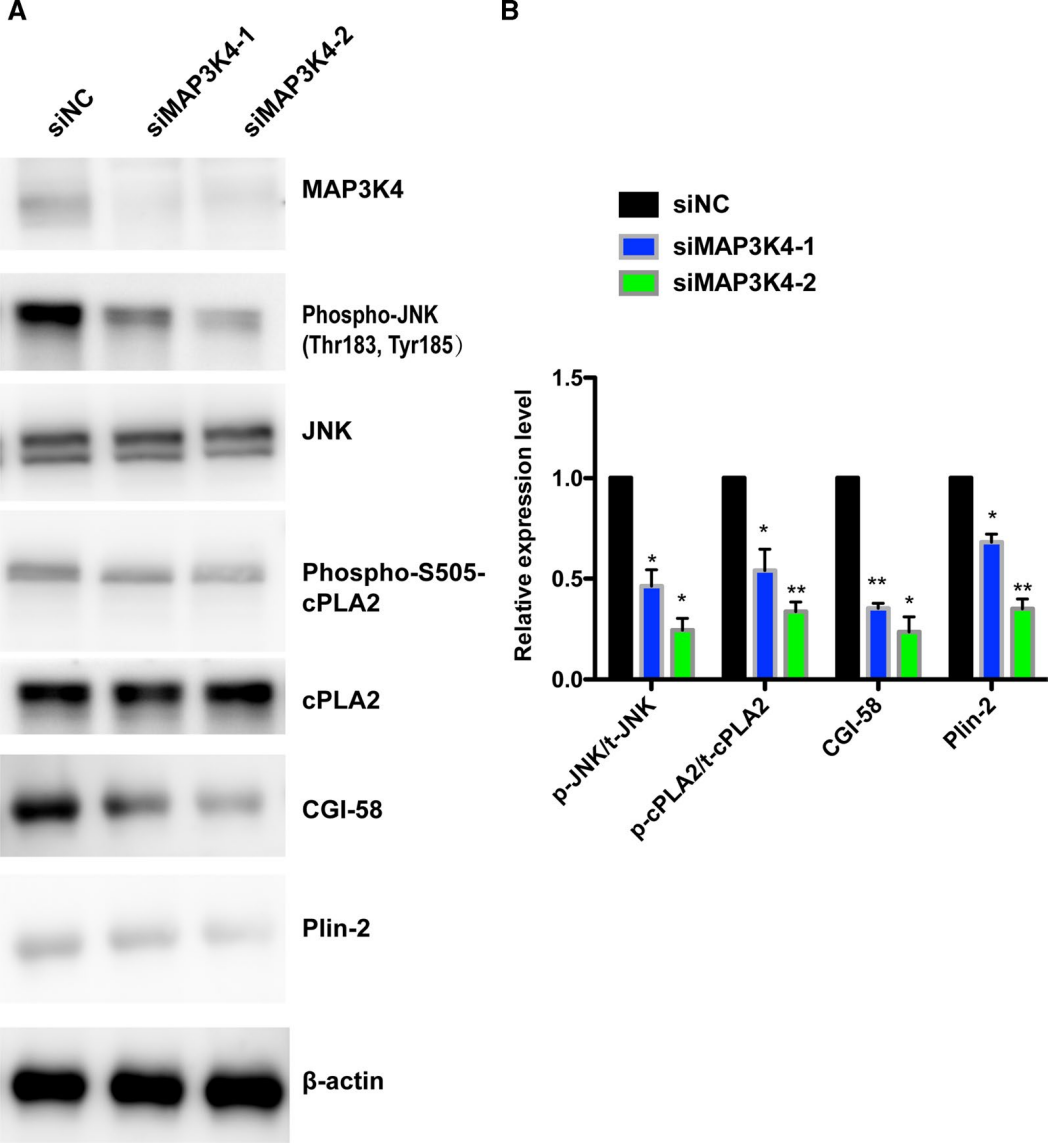
MAP3K4 regulated LDs formation by activation of JNK and cPLA2*.* **A** Western blot analysis of total and phosphorylated JNK and cPLA2, CGI-58 and Plin-2. β-actin is used as a loading control. p-JNK, phosphorylated JNK; t-JNK, total JNK; p-cPLA2, phosphorylated cPLA2; t-cPLA2, total cPLA2. **B** Quantitative results of the relative expression of protein p-JNK/ t-JNK, p-cPLA2/ t-cPLA2, CGI-58 and Plin-2 (*p < 0.05; **p < 0.01)

To elucidate the effects of *MAP3K4* on hepatic lipid metabolism, we downregulated the expression the *MAP3K4* in HepG2 cells by RNA interference, as shown in Fig. 5A. When *MAP3K4* was downregulated, the accumulation of the OA + PA-induced LDs dramatically reduced (Fig. 5A, )B). Knockdown of *MAP3K4* induced the decreased extracellular ALT and AST in HepG2 cells (Fig. 5C, )D). To further confirm the regulation of *MAP3K4* on hepatic LDs formation, LDs markers (CGI-58 and Plin-2) were detected [48]. Knockdown of *MAP3K4* induced the decreased expression of CGI-58 and Plin-2 (Fig. 6A, )B). Activation of JNK and group IVA cPLA2 is a critical process in the biogenesis of LDs [49]. To further explore the underlying mechanism of how *MAP3K4* regulated LDs formation, phosphorylation of JNK and cPLA2 were detected after *MAP3K4* knockdown (Fig. 6A, )B). *MAP3K4* knockdown reduced JNK and cPLA2 activation by inhibiting the phosphorylation of JNK and cPLA2*.*

## Discussions

IN our study, we conducted a comprehensive analysis to identify regulatory factors in NAFLD. 29 hub genes and five MCODE modules were screened using the MCODE algorithm. In the MCODE 5, four of six hub genes (*FABP4*, *SERPINE1*, *GADD45B* and *NAMPT*) has been reported to be associated with lipid metabolism [29–32]. Furthermore, all of these hub genes were densely connected with *MAP3K4.* The study showed that overexpression of *MAP3K4* was associated with abnormal lipid metabolism in NAFLD. Through interference experiments in vitro, our results indicated that *MAP3K4* might be involved in the biogenesis of LDs by regulating the phosphorylation of JNK and cPLA2 in NAFLD.

FABP4, which located in cytoplasm of adipocyte, enhanced lipolysis by interacting with hormone-sensitive lipase [32, 50]. Inhibition of SERPINE1 reduced hepatic expression levels of PCSK9, which is well acknowledged as a regulator of lipid metabolism by impairing receptor-mediated low-density lipoprotein cholesterol (LDL-C) clearance [31, 51]. GADD45B preventsed lipid accumulation by interacting directly with heat shock protein 72 [30]. In high fat diet-induced mouse, upregulation of NAMPT has been shown to improve hepatic lipid homeostasis by reducing triglyceride levels [52–54]. All the above hub genes were densely connected with *MAP3K4.* These results suggested that it was quite reliable to select *MAP3K4* as a candidate gene for further study.

*MAP3K4* is known as *MTK1*, which is an important member of the MAPK signaling pathway [55]. MAPK signaling pathway, consisting of a cluster of protein kinase, plays a crucial role in controlling an abroad of biological activities, including cell proliferation, cell motility, cell survival and death, and gene expression [56] A previous study showed that the JNK cascade was identified as the one governing cPLA2 phosphorylation [49]. In addition, numerous studies have revealed that MAP3K4 is crucial for JNK activation [57]

The JNK signal transduction pathway is crucial for the negative regulation of insulin signaling, which is considered the major contributor to the development of hepatic steatosis [58, 59]. MKK4 and MKK7 are the only two MAP2Ks that activate JNK, and these two upstream MAP2Ks can be activated by MAP3K4 [57]. In addition, connexin and pannexin genes have been demonstrated to be crucial in liver diseases, including NAFLD [60]. In non-alcoholic steatohepatitis, connexin32 exerted a protective effect in connexin32 dominant negative transgenic mice [61]. Connexin32 regulates the expression of JNK and Cdc42. Besides, hepatic inflammation is caused by the activation of pannexin1 in lipoapoptosis [62]. The pannexin1 activation may be mediated by upstream JNK, which can be activated by MAP3K4. These studies suggested that MAP3K4-JNK pathway might be crucial for hepatic lipid metabolism.

cPLA2α, a calcium-dependent enzyme that cleaves fatty acids which is essential for lipid droplet biogenesis, can be activated and phosphorylated by JNK. cPLA2α contributes to the formation of nascent LDs from the endoplasmic reticulum. Phosphorylation at Ser505 is key for cPLA2α enzyme activity and LDs formation [49, 63, 64]. Consistent with previous studies, our findings demonstrated that *MAP3K4* knockdown greatly decreased LDs formation by reducing JNK activation and subsequent cPLA2 phosphorylation at Ser-505 [49, 57]. In addition, due to MAP3K4 control of the insulin-like growth factor 1 receptor, stem cells lacking MAP3K4 kinase activity are less sensitive to insulin stimulation [65]. This offers fresh insight for further research to investigate the function of MAP3K4 in NAFLD.

There are several limitations about the study. It is necessary to do additional research with larger sample sizes to clarify the relationship between the MAP3K4 expression levels of MAP3K4 and clinical indicators of NAFLD severity. The suspected NAFLD patients should also be considered. Additionally, more experiments are needed to further explore the role of *MAP3K4* in NAFLD in vivo and in vitro. Finally, the roles of the other 28 hub genes in NAFLD, and their underlying mechanisms deserved further investigation.

## Conclusion

In conclusion, we discovered several regulatory genes involved in the hepatic lipid metabolism of NAFLD. The present study demonstrated that elevated expression of *MAP3K4* causes abnormal accumulation in NAFLD by activating JNK and cPLA2. The MAP3K4-JNK-cPLA2 pathway may play a crucial role in lipogenesis of NAFLD.

## Supplementary Information

**Additional file 1: Table S1.** Clinical Characteristics of NAFLD patients and controls.

**Additional file 2: Table S2.** RNA-seq data of liver tissues of NAFLD patients and controls.

**Additional file 3: Table S3.** Results of GO enrichment on differentially expressed genes from RNA-seq data.

**Additional file 4: Table S4.** The top 20 significantly KEGG pathways of RNA-seq data.

**Additional file 5: Table S5.** Results of KEGG enrichment on differentially expressed genes from GSE159676 datasets.

**Additional file 6: Table S6.** Results of GO enrichment on differentially expressed genes from GSE159676 datasets.

**Additional file 7: Table S7.** Differentially expressed genes that appeared in both RNA-seq data and the GSE159676 datasets

**Additional file 8: Figure S1.** PPI network based on the analysis of 134 overlapping genes using Cytoscape.

**Additional file 9: Table S8.** The top 10 hub genes rank in Betweenness algorithm of CytoHubba.

## Data Availability

The datasets during and/or analyzed during the current study available from the corresponding author on reasonable request.

## Abbreviations

ALT: Alanine aminotransferase
AST: Aspartate aminotransferase
cPLA2: Cytosolic phospholipase A2
IR: Insulin resistance
JNK: Jun N-terminal kinase
*MAP3K4*: Mitogen-activated protein kinase kinase kinase 4
NAFLD: Non-alcoholic fatty liver disease
NAS: NAFLD activity score
NASH: Non-alcoholic steatohepatitis
LDs: Lipid droplets
MCODE: Molecular complex detection
OA: Oleic acid
PA: Palmitic acid
PPI: Protein–protein interaction
RNA-seq: RNA sequencing
