# Supplementary material for: Identification of MAP3K4 as a novel regulation factor of hepatic lipid metabolism in non-alcoholic fatty liver disease
Source: J Transl Med. 2022 Nov 14;20:529. doi: 10.1186/s12967-022-03734-8 (PMC9664664; doi:10.1186/s12967-022-03734-8)
Supplement: Supplementary file 1 — Additional file 1: Table S1. Clinical Characteristics of NAFLD patients and controls. [file 12967_2022_3734_MOESM1_ESM.docx]

**Table S1. Clinical Characteristics of NAFLD and control groups.**

| **NO** | **Sex** | | **Age**  **(year)** | | **BMI**  **(kg/m^2^)** | | | **WC**  **(cm)** | | **ALT**  **(U/L)** | **AST (U/L)** | **ALP**  **(U/L)** | **γ-GT (U/L)** | **TC**  **(mmol/L)** | **Tri**  **(mmol/L)** | **HDL-C**  **(mmol/L)** | **LDL-C**  **(mmol/L)** | **FBG**  **(mmol/L)** | **UA**  **(umol/L)** | **HbA1c**  **(％)** | **HOMA-IR** | **MetS** | **Diabetes** | **Hypertension** | **NAS** |
| --- | --- | --- | --- | --- | --- | --- | --- | --- | --- | --- | --- | --- | --- | --- | --- | --- | --- | --- | --- | --- | --- | --- | --- | --- | --- |
|  | | **NAFLD** | |  | |  |  | |  |  |  |  |  |  |  |  |  |  |  |  |  |  |  |  |  |
| 1 | M | | 54 | | 27.8 | | | 96 | | 27.8 | 35.5 | 47.4 | 38.8 | 5.10 | 1.68 | 1.21 | 2.87 | 5.10 | 437.14 | 5.90 | 1.5 | - | - | + | 4 |
| 2 | M | | 42 | | 37.2 | | | 130 | | 183.7 | 65.5 | 110.5 | 119.4 | 6.67 | 2.45 | 1.14 | 3.97 | 5.50 | 543.64 | 5.83 | 1.7 | + | - | + | 6 |
| 3 | M | | 40 | | 30.5 | | | 110 | | 49.4 | 26.5 | 93.7 | 78.4 | 6.62 | 2.07 | 0.95 | 4.18 | 6.20 | 518.76 | 7.86 | 1.8 | + | + | + | 5 |
| 4 | M | | 37 | | 30.4 | | | 109 | | 25.0 | 37.5 | 56.7 | 44.7 | 5.68 | 1.98 | 0.92 | 2.75 | 6.80 | 481.70 | 7.21 | 1.8 | + | + | - | 5 |
| 5 | M | | 50 | | 22.2 | | | 91 | | 25.3 | 28.6 | 47.6 | 48.6 | 6.02 | 1.93 | 1.04 | 3.65 | 7.30 | 506.71 | 6.70 | 1.9 | + | + | + | 5 |
| 6 | M | | 45 | | 32.7 | | | 130 | | 56.4 | 35.6 | 67.8 | 73.4 | 5.45 | 1.86 | 0.95 | 3.41 | 5.80 | 387.43 | 7.33 | 1.6 | + | + | - | 5 |
| 7 | M | | 46 | | 22.5 | | | 91 | | 100.7 | 55.9 | 98.4 | 113.4 | 4.98 | 1.54 | 1.26 | 2.78 | 6.70 | 456.78 | 7.42 | 1.8 | - | + | + | 4 |
|  | | **Control** | |  | |  |  | |  |  |  |  |  |  |  |  |  |  |  |  |  |  |  |  |  |
| A | M | | 48 | | 23.1 | | | 92 | | 11.9 | 12.3 | 48.7 | 37.4 | 4.61 | 1.56 | 1.31 | 2.54 | 4.90 | 367.1 | 3.80 | 1.0 | - | - | - | 0 |
| B | M | | 53 | | 23.9 | | | 92 | | 25.0 | 25.4 | 44.7 | 67.5 | 4.43 | 1.64 | 1.41 | 2.78 | 5.80 | 407.5 | 4.20 | 0.9 | - | - | - | 1 |
| C | M | | 37 | | 25.4 | | | 95 | | 30.7 | 29.8 | 92.7 | 42.7 | 4.86 | 1.44 | 1.26 | 2.76 | 4.30 | 478.7 | 4.37 | 1.0 | - | - | - | 2 |
| D | M | | 31 | | 21.5 | | | 88 | | 49.0 | 31.2 | 51.7 | 78.6 | 5.74 | 1.87 | 1.56 | 2.86 | 4.90 | 367.3 | 3.58 | 1.0 | - | - | + | 3 |
| E | M | | 54 | | 23.9 | | | 93 | | 8.7 | 15.8 | 51.3 | 27.8 | 4.51 | 1.34 | 1.54 | 2.47 | 6.30 | 398.7 | 6.10 | 1.3 | - | + | - | 2 |
| F | M | | 49 | | 24.2 | | | 94 | | 33.7 | 29.7 | 76.3 | 34.1 | 4.94 | 1.68 | 1.47 | 2.73 | 4.90 | 373.2 | 2.75 | 1.1 | - | - | - | 1 |
| G | M | | 52 | | 16.3 | | | 78 | | 35.7 | 43.3 | 47.8 | 65.8 | 4.57 | 1.68 | 1.45 | 2.55 | 4.50 | 417.2 | 4.42 | 1.0 | - | - | + | 1 |
| H | M | | 29 | | 22.1 | | | 90 | | 38.5 | 24.8 | 37.4 | 44.5 | 4.60 | 1.37 | 1.36 | 2.89 | 4.60 | 376.8 | 4.47 | 1.0 | - | - | - | 0 |
| I | M | | 48 | | 23.7 | | | 92 | | 24.6 | 22.6 | 61.8 | 55.4 | 4.93 | 1.57 | 1.27 | 2.64 | 5.50 | 421.7 | 5.34 | 1.0 | - | - | - | 1 |

**Abbrebiations**: BMI, body mass index; WC, waist circumference; ALT, alanine aminotransferase; AST, aspartate aminotransferase; ALP, alkaline phosphatase; γ-GT, γ-glutamyl transpeptidase; TC, total cholesterol; Tri, Triglycerides; HDL-C, high-density lipoprotein cholesterol; LDL-C, low-density lipoprotein cholesterol; FBG, fasting blood glucose; HbA1c, Hemoglobin A1C; HOMA-IR, Homeostasis Model Assessment of Insulin Resistance; UA, uric acid; MetS, metabolic syndrome; NAS, NAFLD activity score.
