# Supplementary figures and images for: Identification of MAP3K4 as a novel regulation factor of hepatic lipid metabolism in non-alcoholic fatty liver disease
Source: J Transl Med. 2022 Nov 14;20:529. doi: 10.1186/s12967-022-03734-8 (PMC9664664; doi:10.1186/s12967-022-03734-8)

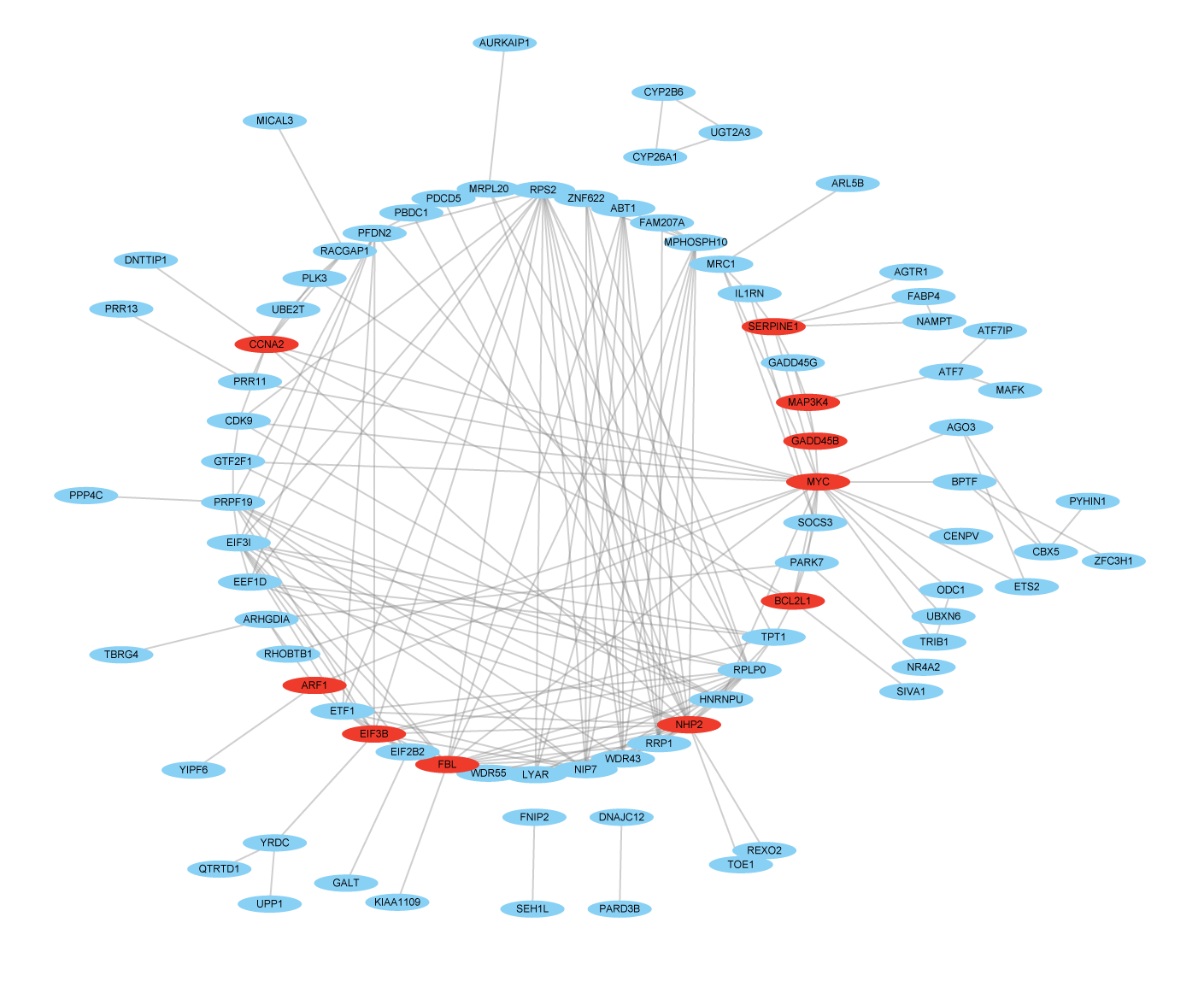

Supplement: Supplementary file 8 — Additional file 8: Figure S1. PPI network based on the analysis of 134 overlapping genes using Cytoscape. [file 12967_2022_3734_MOESM8_ESM.tif]
