# Supplementary material for: Identification of MAP3K4 as a novel regulation factor of hepatic lipid metabolism in non-alcoholic fatty liver disease
Source: J Transl Med. 2022 Nov 14;20:529. doi: 10.1186/s12967-022-03734-8 (PMC9664664; doi:10.1186/s12967-022-03734-8)
Supplement: Supplementary file 9 — Additional file 9: Table S8. The top 10 hub genes rank in Betweenness algorithm of CytoHubba. [file 12967_2022_3734_MOESM9_ESM.docx]

**The top 10 Hub genes of overlapping genes**

| **Gene Symbol** | **Expression** | **Score** | **Rank** |
| --- | --- | --- | --- |
| MYC | down | 3351.030152 | 1 |
| FBL | down | 1432.834917 | 2 |
| NHP2 | down | 1163.808592 | 3 |
| CCNA2 | up | 884.4311977 | 4 |
| GADD45B | down | 602 | 5 |
| SERPINE1 | down | 562.6768467 | 6 |
| EIF3B | down | 545.8761955 | 7 |
| MAP3K4 | up | 444 | 8 |
| ARF1 | down | 374.144825 | 9 |
| BCL2L1 | down | 367.9329797 | 10 |
